# Identification of Common and Distinct Pathways in Inflammatory Bowel Disease and Colorectal Cancer: A Hypothesis Based on Weighted Gene Co-Expression Network Analysis

**DOI:** 10.3389/fgene.2022.848646

**Published:** 2022-03-31

**Authors:** Afshin Derakhshani, Darya Javadrashid, Nima Hemmat, Antoine Dufour, Antonio Giovanni Solimando, Mahdi Abdoli Shadbad, Pascal H. G. Duijf, Oronzo Brunetti, Nicola Silvestris, Behzad Baradaran

**Affiliations:** ^1^ Department of Biochemistry and Molecular Biology, University of Calgary, Calgary, AB, Canada; ^2^ Immunology Research Center, Tabriz University of Medical Sciences, Tabriz, Iran; ^3^ Departments of Physiology and Pharmacology, University of Calgary, Calgary, AB, Canada; ^4^ McCaig Insitute, Hotchkiss Brain Institute and Snyder Institute for Chronic Diseases, University of Calgary, Calgary, AB, Canada; ^5^ Department of Biomedical Sciences and Human Oncology, School of Medicine, Aldo Moro University of Bari, Bari, Italy; ^6^ Student Research Committee, Tabriz University of Medical Sciences, Tabriz, Iran; ^7^ School of Biomedical Sciences, Faculty of Health, Queensland University of Technology, Brisbane, QLD, Australia; ^8^ Centre for Data Science, Queensland University of Technology, Brisbane, QLD, Australia; ^9^ Translational Research Institute, University of Queensland Diamantina Institute, The University of Queensland, Brisbane, QLD, Australia; ^10^ Medical Oncology Unit, IRCCS Istituto Tumori Giovanni Paolo II, Bari, Italy; ^11^ Medical Oncology Unit, Department of Human Pathology “G. Barresi” , University of Messina, Messina, Italy; ^12^ Department of Immunology, Faculty of Medicine, Tabriz University of Medical Sciences, Tabriz, Iran

**Keywords:** colorectal cancer, inflammatory bowel disease, pathogenesis, systems biology, WGCNA

## Abstract

Patients with inflammatory bowel disease (IBD), including ulcerative colitis and Crohn’s disease, are at higher risk to develop colorectal cancer (CRC). However, the underlying mechanisms of this predisposition remain elusive. We performed in-depth comparative computational analyses to gain new insights, including weighted gene co-expression network analysis (WGCNA) and gene ontology and pathway enrichment analyses, using gene expression datasets from IBD and CRC patients. When individually comparing IBD and CRC to normal control samples, we identified clusters of highly correlated genes, differentially expressed genes, and module-trait associations specific for each disease. When comparing IBD to CRC, we identified common hub genes and commonly enriched pathways. Most notably, IBD and CRC share significantly increased expression of five genes (*MMP10*, *LCN2*, *REG1A*, *REG3A,* and *DUOX2*), enriched inflammatory and neutrophil activation pathways and, most notably, highly significant enrichment of IL-4 and IL-13 signaling. Thus, our work expands our knowledge about the intricate relationship between IBD and CRC development and provides new rationales for developing novel therapeutic strategies.

## 1 Introduction

Colorectal cancer (CRC) is one of the leading causes of cancer-related morbidity and mortality globally (Rawla et al., 2019). It is the third most common cancer and the fourth most common cause of cancer-related deaths worldwide (Haggar and Boushey, 2009). Despite considerable advances in the treatment of CRC patients over the past decades, treatment response rates remain variable. Thus, there is a need to develop new therapies with higher efficacies and minimal side effects (Derakhshani et al., 2021a).

Inflammatory bowel disease (IBD) is a chronic multifactorial relapsing-remitting disease, and two main subtypes are Crohn’s disease (CD) and ulcerative colitis (UC). In genetically predisposed individuals, incompletely identified environmental stimuli result in an aberrant immune-driven inflammatory response directed towards altered gut microbiota (Yalchin et al., 2021).

There is a close association between chronic inflammation and CRC development (Balkwill and Mantovani, 2001; Korniluk et al., 2017). The dysregulated immune responses in IBD are considered risk factors for CRC development (Kim and Chang, 2014; Zhang et al., 2017). CRC risk in patients with IBD was assessed in the first comprehensive meta-analysis in 2001 by Eaden et al., and their results showed a 2% risk of CRC at 10 years following UC diagnosis, an 8 percent risk at 20 years, and an 18 percent risk at 30 years, with an overall CRC prevalence of 3.7 percent. Patients with UC have a 1.7-fold increased risk of developing CRC, with an overall adjusted hazard ratio of 1.66 (95 percent confidence interval: 1.57–1.76), and a 1.6-fold increased risk of dying from CRC, when compared with the general population, according to the most extensive population study conducted to date (*n* = 96,447) (Eaden et al., 2001).

Additional risk factors include microbes, immunological responses, genetic mutations, and lifestyle/environmental triggers, yet the driving mechanism(s) of CRC remains uncharacterized mainly (Kaistha and Levine, 2014). Indeed, a better understanding of the molecular mechanisms of chronic inflammation could help identify novel drug targets for more personalized therapy for each patient (Ogunwobi et al., 2020).

Systems biology approaches, including transcriptomics, proteomics, and metabolomics, offer an unbiased survey of individual CRC patients (Derakhshani et al., 2020; Hemmat et al., 2020). Network-based approaches comprise reliable methods to identify specific pathways that could be regulating CRC. Since CRC is rarely caused by a single gene mutation but rather by disruptions in intricate gene networks, network-based approaches have provided a plethora of new opportunities to study the pathogenesis of cancer and to develop new cancer therapeutic approaches (Barabási et al., 2011).

Weighted gene co-expression network analysis (WGCNA) is an effective network-based approach that identifies clusters–or modules–of highly correlated genes. WGCNA is designed to analyze gene expression datasets by quantifying the associations between independent gene pairs and the degree to which these genes share the same neighbors (Hemmat et al., 2020). This tool is particularly suitable for identifying new potential biomarkers or therapeutic target genes, a strategy that has been successfully applied to a range of cancers (Derakhshani et al., 2020; Liao et al., 2020). However, WGCNA has not yet been used to study the link between IBD and CRC. Therefore, we used WGCNA to identify differentially expressed genes and enriched pathways in IBD and CRC in our current study. The significantly changing genes and pathways that we identified will further our knowledge of the relationship between IBD and CRC development.

## 2 Materials and Methods

### 2.1 Microarray Datasets and Processing

The GSE110224 and GSE4183 microarray datasets were downloaded from the GEO database (https://www.ncbi.nlm.nih.gov/geo/). The GSE110224 dataset was based on GPL570 [HG-U133_Plus_2] Affymetrix Human Genome U133 Plus 2.0 Array with 34 samples (17 patients with colorectal cancer and 17 matched adjacent normal tissue samples). All samples of this dataset were used for downstream analyses (*n* = 34). The GSE4183 dataset was created on the GPL570 [HG-U133_Plus_2] Affymetrix Human Genome U133 Plus 2.0 Array and contained 53 samples, including 15 patients with CRC, 15 with adenoma, 15 IBD tissue samples, and eight healthy normal control tissue samples. Eight control samples and the 15 IBD samples of this dataset were used for analyses (*n* = 23; Supplementary Table S1). The raw data were corrected, quantile-normalized, and probe IDs were converted to gene symbols. If there were multiple probes for genes, probe signals for the same gene were collapsed by averaging to yield a single expression level per gene per sample. Gene symbols were filtered across all samples through their variance. Only genes with variances ranked in the top 5,000 were selected for subsequent analyses.

### 2.2 Construction of Co-Expression Modules of CRC and IBD

Co-expression networks for gene expression data of patients and the control group were reconstructed using the R (http://www.r-project.org/index.html) and R/Bioconductor packages (Gentleman et al., 2004), such as *WGCNA* (Langfelder and Horvath, 2008). Briefly, the matrix of the gene expression profiles was converted into the matrix of pairwise gene similarity according to the Pearson test, followed by conversion into a matrix of adjacency. According to the already represented scale-free gene co-expression topological algorithm, when the β value is considered 12 and 7 (GSE110224 and GSE4183, respectively), the adjacency matrix met the scale-free topology criteria. Then, a topological overlap matrix (TOM) and a “dissimilarity” matrix (dissTOM) were created using TOM similarity and dissimilarity modules, based on the correlation of the pairwise gene-co-expression and according to Eq. I.

dissTOM=1−TOM (I)

Finally, the clusters of highly interconnected genes were created with a minimum module size of 30 genes and a cut height of 0.001 (Horvath, 2011).

### 2.3 Construction of Module-Trait Relationships and Hub Genes of CRC and IBD

To identify modules significantly related to the evaluated clinical trait, the expression profiles of each module were summarized via their module eigengene (ME) as the eigenvector correlated to the first principal component of the expression matrix. The gene significance (GS) values were used for measuring individual gene associations with CRC and IBD. Also, module membership (MM) was defined as the correlation of the ME and the gene expression profile for each module. When the GS and MM were highly correlated (determined by Pearson correlation analysis), the most significant (central) elements in the modules were also closely associated with the trait (Zhang and Horvath, 2005). The top-ranked significant module genes from CRC were compared with the most significant IBD module genes using the freely available *Venny* tool (version 2.1) (http://bioinfogp.cnb.csic.es/tools/venny/). Then, Co-expression networks of shared gene modules between CRC and IBD were then constructed using GeneMANIA (https://genemania.org/).

### 2.4 Identification of DEGs and Biological Pathways for CRC and IBD

Identification of DEGs among patients and normal samples was carried out using a volcano plot, indicating the upregulated and downregulated genes in CRC and IBD samples. DEGs were called if they met two criteria: (1) $\left|{\text{log}}_{2}\left(\text{FC}\right)\right|\geq 2$ and (2) $p_{adjust}<0.01$ . Herein, FC represents the fold change in gene expression and $p_{adjust}$ denotes the Benjamini and Hochberg-adjusted *p-value* to compensate for multiple hypothesis testing. To investigate the biological functions and pathways of selected DEGs and gene modules, functional gene ontology (GO) terms and Kyoto Encyclopedia of Genes and Genomes (KEGG) pathway enrichment analyses were performed, and results were visualized using the *ClueGO* v2.5.7 and *CluePedia* v1.5.7 plugins of Cytoscape v3.8.1 [18]. Enriched ontological terms and pathways with a threshold of Benjamini-adjusted *p*-value < 0.000001 were evaluated. Alternatively, pathway analysis was performed using *Reactome* (Jassal et al., 2020) and Benjamini-adjusted *p*-values.

### 2.5 Identification of DEGs for CRC and IBD

The top significant module genes of each dataset and DEGs were selected for comparison with other datasets. Common genes were chosen for further analysis using the *Venny* tool (version 2.1) software available freely at (http://bioinfogp.cnb.csic.es/tools/venny/).

The *pheatmap* visualization *R* package (https://CRAN.R-project.org/package=pheatmap) was used to show gene expression patterns between IBD and normal tissues. Additionally, the approach was used to demonstrate the gene expression patterns between CRC and normal samples. Then the shared genes were visualized.

### 2.6 External Validation of Common Genes

For CRC, external validation of common IBD and CRC genes was carried out by using The Cancer Genome Atlas (TCGA) dataset in the UCSC Xena browser (https://xenabrowser.net/). The gene expression levels in primary tumors, normal solid tissue, recurrent tumor, and metastatic tumor tissues were extracted from the TCGA-COAD dataset using the UCSC Cancer Browser. Additionally, to identify the overlap between our findings and independent studies and validate our results using various microarray and RNA-seq data, GEO2R tool (https://www.ncbi.nlm.nih.gov/geo/geo2r/; R 3.2.3, Biobase 2.30.0, GEOquery 2.40.0, limma 3.26.8) and edgeR package (Robinson et al., 2010) were applied in the current study (Supplementary Table S1).

### 2.7 Statical Analyses

R versions (3.2.0 and 4.02) and a range of R packages for statistical analyses, such as WGCNA, annotate, Limma, Affy, AgiMicroRna, ggplot2, edgeR, and pheatmap were used. The statistical tests used are specified in the main text, and Benjamini-adjusted *p*-values were used to compensate for multiple-hypothesis testing.

## 3 Results

### 3.1 Identification of Modules in CRC and IBD Samples

**FIGURE 1:**
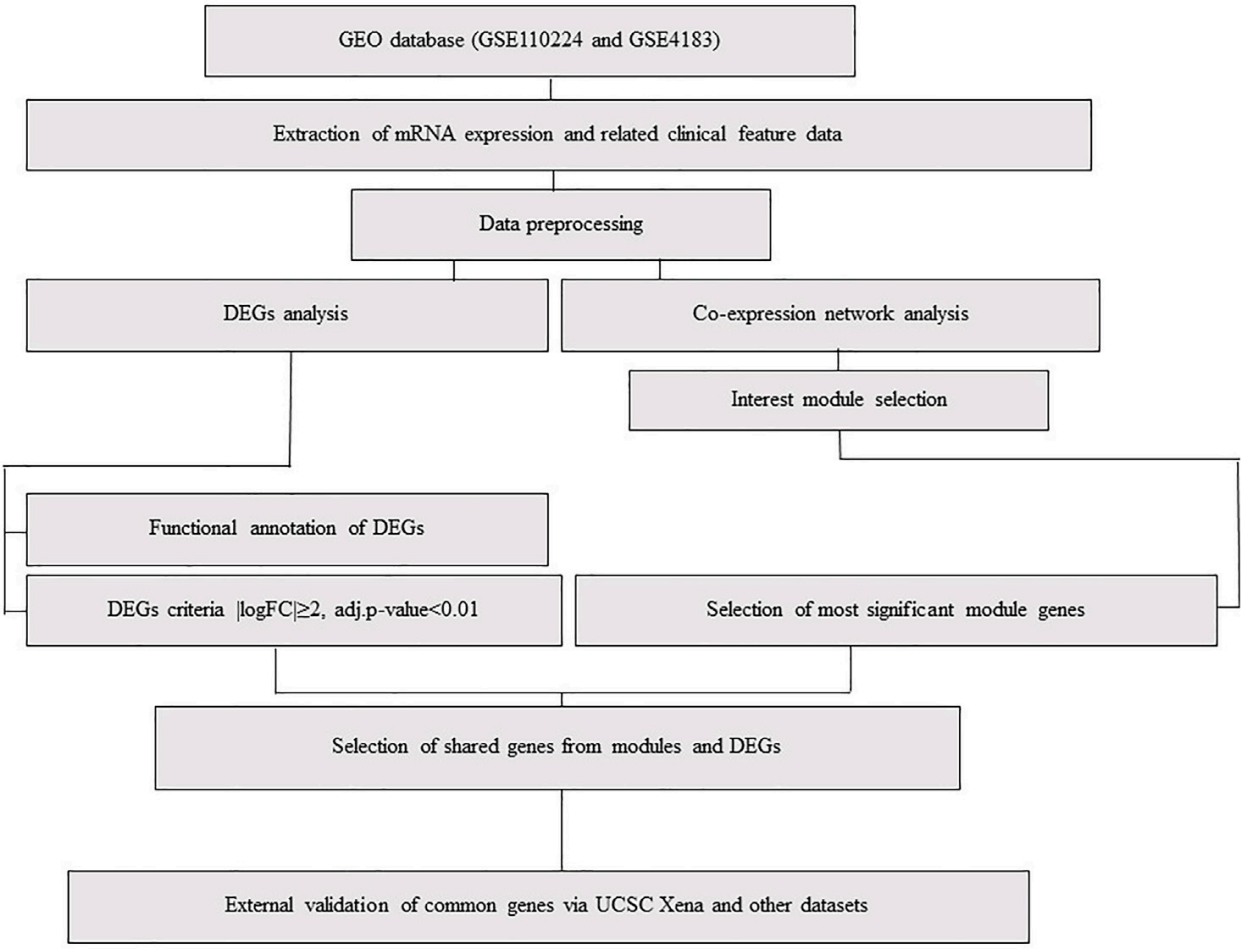
Flowchart, including data preparation, processing, and analysis of CRC and IBD datasets. The CRC dataset (GSE110224) includes 17 CRC samples and 17 matched normal samples from each patient (*n* = 34). The IBD dataset (GSE4183) includes eight normal colon tissue samples and 15 colon IBD samples (*n* = 23).

To investigate gene expression’s overlap and differences between IBD and CRC, we used two microarray datasets: GSE110224 and GSE4183 (Figure 1). For CRC, GSE110224 includes 17 CRC samples and 17 matched adjacent normal tissue samples. For IBD, GSE4183 contains normal colon tissue samples (*n* = 8) and IBD samples (*n* = 15). First, we performed a clustering analysis, indicating that normal, CRC, and IBD samples clustered together across the two datasets (Supplementary Figure S1). Then, specific soft-threshold power was selected for each dataset. A weighted co-expression network was constructed for CRC and IBD, using the normal samples in each of these datasets as controls (Supplementary Figure S2; see Methods for details). We constructed clustering dendrograms for module illustration with different colors (Supplementary Figure S3). The numbers of genes included in each module in each dataset are shown in Supplementary Table S1 and Supplementary Table S2. The genes in the gray “module” were not classified into any modules, as they failed to classify as a distinct co-expression module*.*

### 3.2 Identification of Module-Trait Associations

We evaluated module-module correlations and eigengenes for each module to determine how modules were associated with the disease. Our weighted gene co-expression network analysis (WGCNA) revealed that the blue module, derived from the CRC dataset (GSE110224), had a negative correlation. This was the most significant module in the CRC dataset. However, the turquoise module in the GSE4183 showed a positive correlation (Supplementary Figure S4, Supplementary Table S1, and Supplementary Table S2). The correlation between module membership (MM) and gene significance (GS) of the selected modules was highly statistically significant (*R* ≥ 0.95, *p* < 10^−200^ for blue module and R ≥ 0.93, *p* < 10^−200^ for the turquoise module; Pearson correlation; Supplementary Figure S5). The illustrated genes in each module are highly significant and are most correlated with IBD and CRC.

### 3.3 Mutual Hub Genes From the Most Significant Modules Identify Common Pathways

**FIGURE 2:**
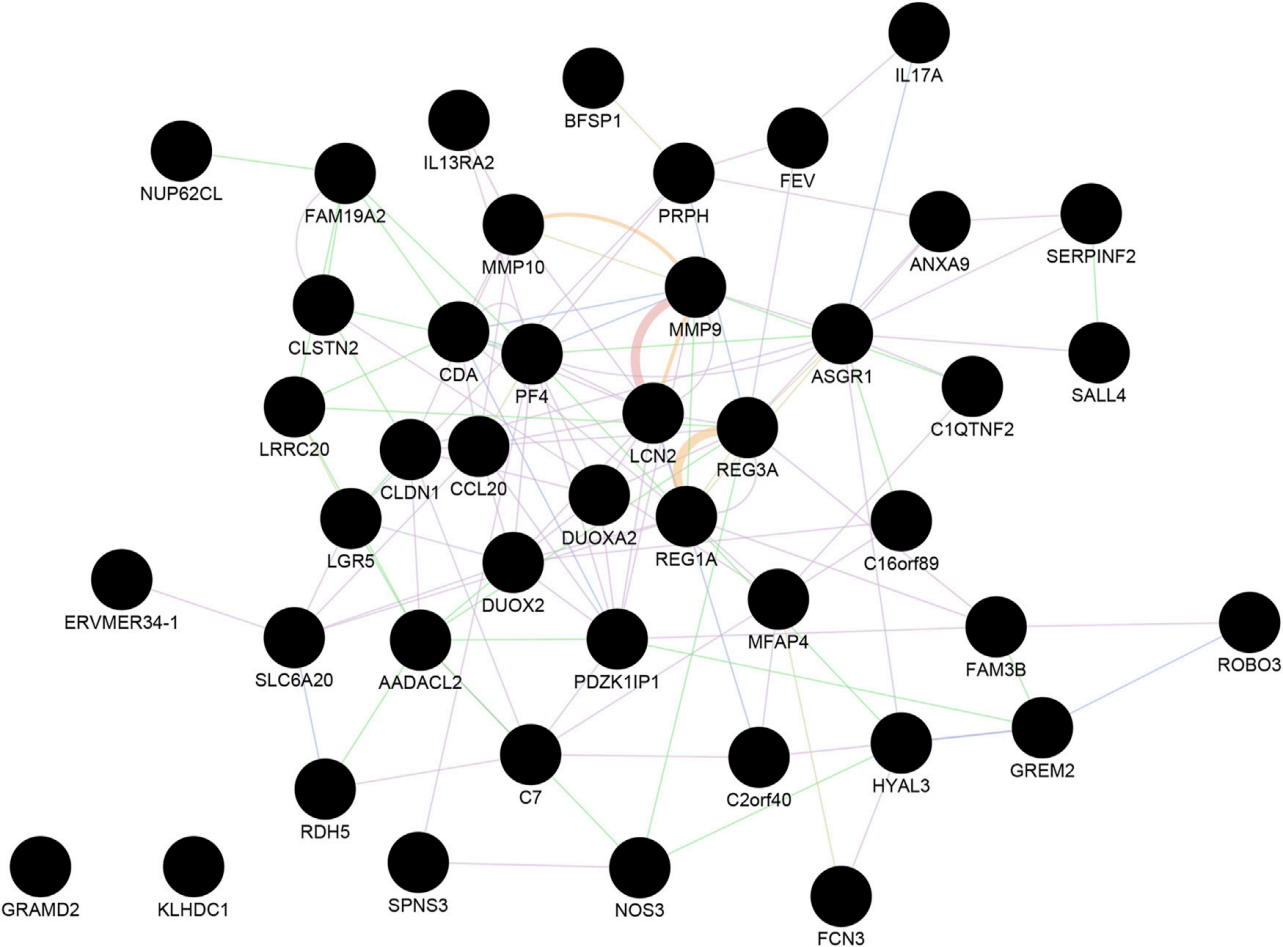
The shared gene modules between CRC and IBD. A total of 48 shared hub genes (cancer compared with normal tissue; IBD compared with normal samples) were identified and imported to GeneMANIA to construct a co-expression and gene interaction network. Each circle represents a gene. Each edge (line) represents interactions between two genes. Multiple lines correspond to multiple sources, as indicated by the color coding. See also Supplementary Tables S3, S4 for additional information.

Next, we identified the most significant shared gene modules in the two datasets by assessing the overlap of the identified genes. Altogether, 201 mutual gene modules were identified between the two datasets. This network comprised 48 genes (Supplementary Table S3, Figure 2). Pathway analysis using Reactome identified IL-4 and IL-13 signaling as significantly enriched (adjusted *p*-value = 0.0003) (Supplementary Table S4).

### 3.4 Identification of Genes Differentially Expressed in CRC and IBD Datasets

**FIGURE 3:**
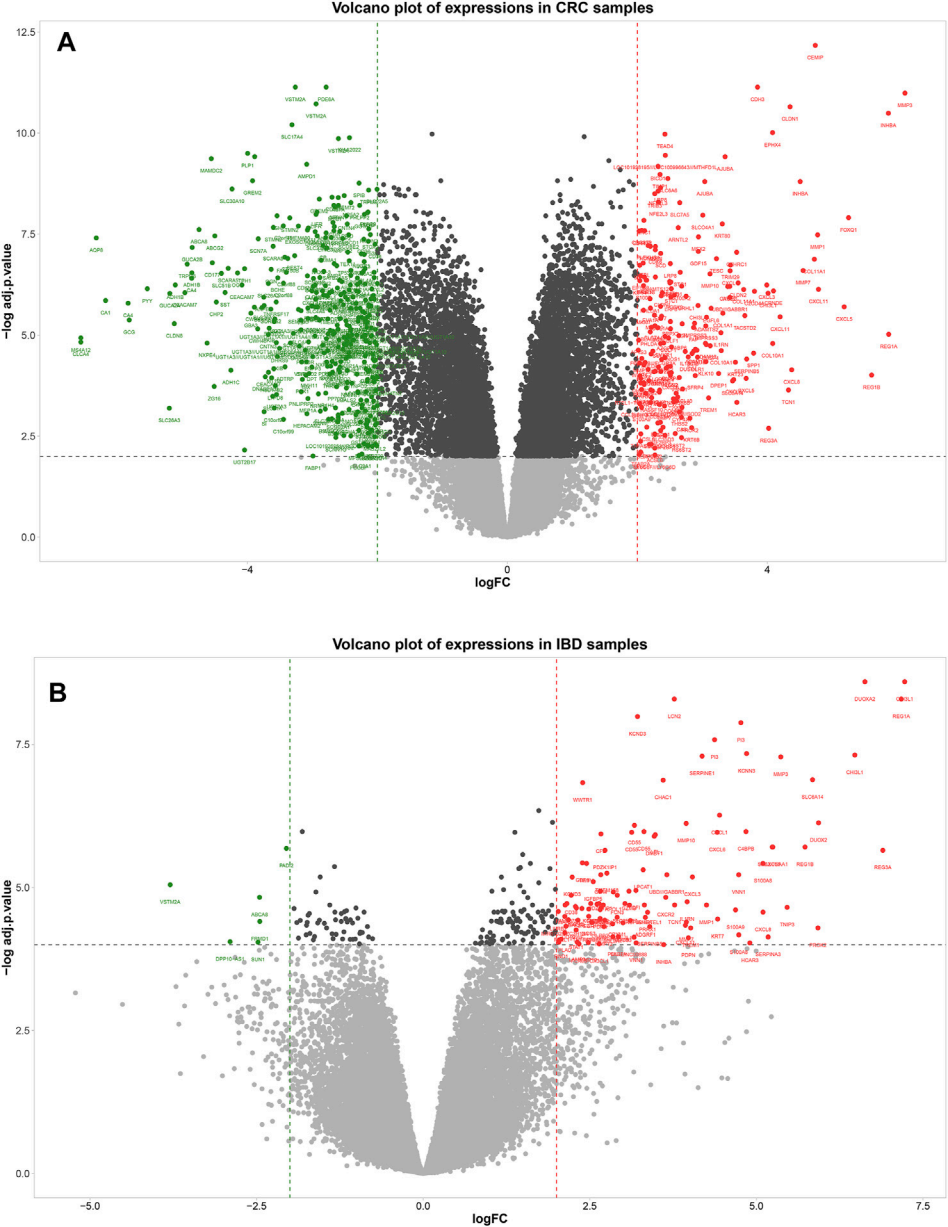
Volcano plot showing differentially expressed genes between tumors normal tissue adjacent to the tumors **(A)** and IBD and normal samples **(B)**. In the volcano plot, the vertical axis (*y*-axis) is the mean value of -log_10_ (adj *p*-value), and the horizontal axis (*x*-axis) represents the value of log(FC). Red dots denote the upregulated genes; green dots represent the down-regulated genes. [**(A)**: CRC dataset GSE110224 and **(B)**: IBD dataset GSE4183]. The cut-off criteria for DEG analysis in CRC dataset and IBD dataset were set as |logFC| ≥ 2, adj. *p*-value < 0.01, and |logFC| ≥ 2, adj. *p*-value < 0.0001, respectively. Genes plotted as grey dots do not meet these criteria.

A total of 573 genes were identified as differentially expressed genes (DEGs) in the CRC samples using thresholds of adjusted *p*-value < 0.01 and |logFC| ≥ 2, including 201 upregulated and 372 down-regulated genes in the tumor compared to the adjacent tissue. In the IBD dataset, 116 genes were detected as DEGs using the same thresholds, including 110 upregulated and six down-regulated genes in IBD compared to normal samples. The volcano plots in Figure 3 show the upregulated and downregulated genes in CRC and IBD samples. Both DEGs shared the most important genes involved in inflammation and cancer, such as *CXCL3*, *MMP7*, *MMP3 INHBA*, *CXCL2*, *CEMIP*, *DUOX2*, *GZMB*, *CXCL1*, *CXCL11*, *CXCL8*, *REG1A*, *VSTM2A*, *REG1B*, *CXCL5, CXCL6*, *REG3A*, *MMP10*, *SLC6A14*, *MMP1*, *LCN2, S100A9,* etc.

### 3.5 GO and KEGG Analysis of DEGs

**FIGURE 4:**
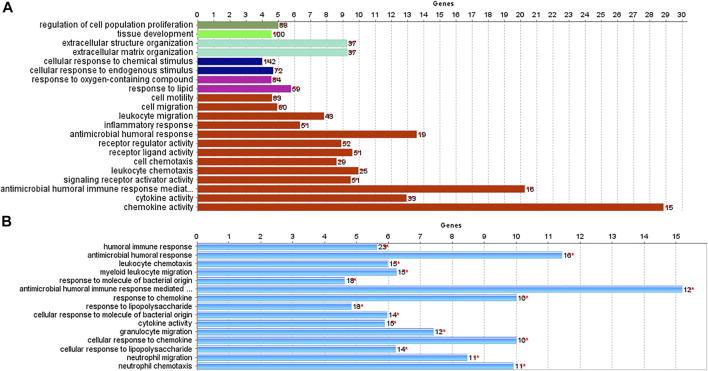
Biological processes and pathways identified in CRC and IBD using their differentially expressed genes (DEGs). The analysis was performed using the ClueGO and CluePedia tools. [**(A)**: CRC dataset GSE110224; **(B)**: IBD dataset GSE4183].

We used the *ClueGO* and *CluePedia* tools to investigate the biological processes and pathways of identified DEGs. Biological functions and pathways associated with CRC were receptors to chemokine activity, antimicrobial humoral immune response mediated by an antimicrobial peptide, extracellular matrix organization, and extracellular structure organization. For IBD, enriched pathways included inflammatory response, antimicrobial humoral response, and neutrophil activation. As demonstrated in Figure 4, antimicrobial humoral response and cytokine activation were shared pathways in CRC and IBD datasets, further supporting the key role of inflammation in CRC and IBD.

### 3.6 Common Genes Detection in all Datasets and Illustration by Heatmap

**FIGURE 5:**
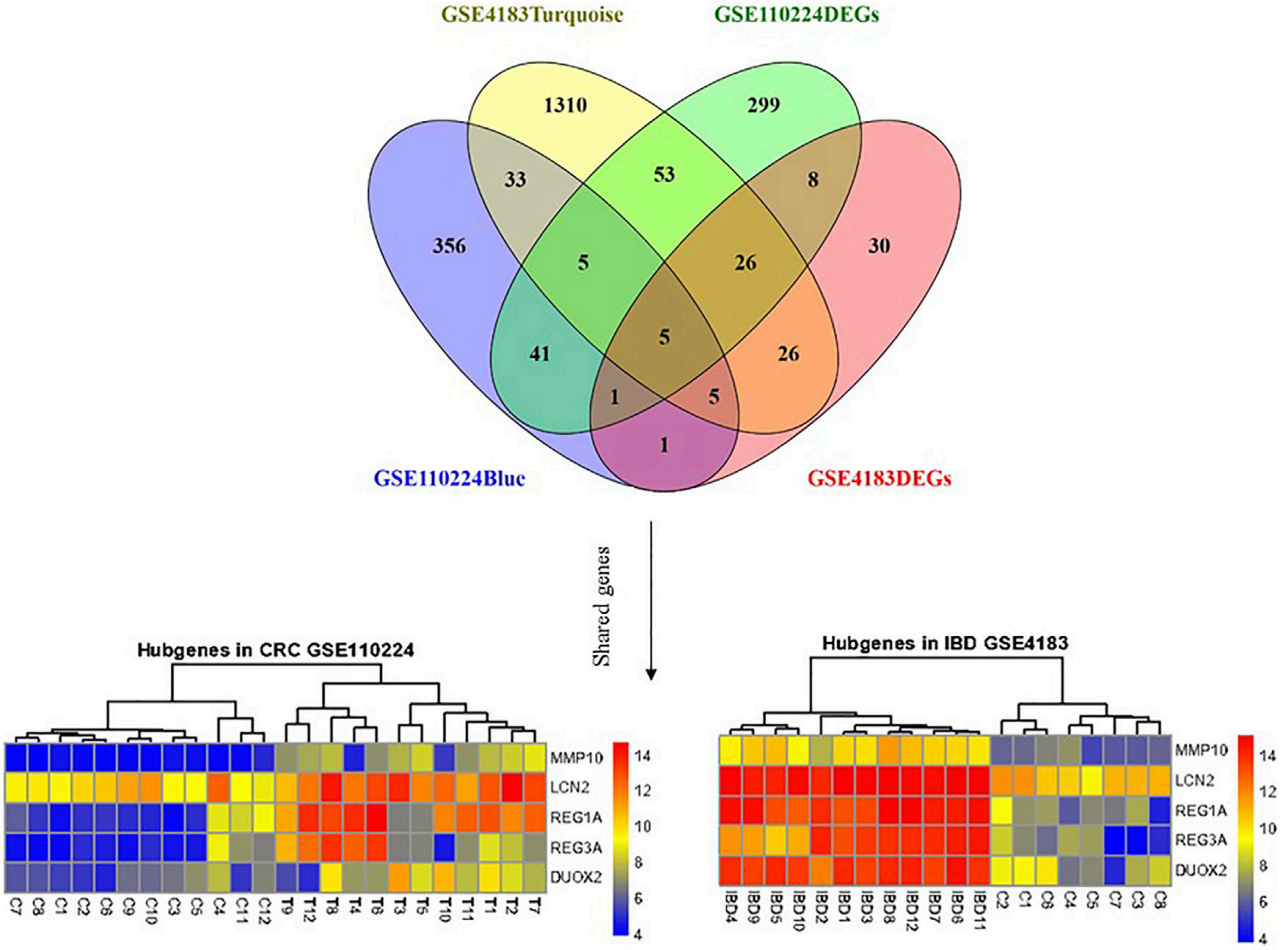
Shared gene expression in CRC and IBD patients compared to normal control tissues. The left side shows the varied expression values between CRC tissues in comparison with adjacent normal tissue, and the right plot illustrates the expression of candidate genes between IBD and corresponding normal control tissues. The upregulated and down-regulated genes are shown as red and blue tiles, respectively.

*MMP10, LCN2, REG1A, DUOX2,* and *REG3A* genes were considered mutual genes between the two primary analyzed datasets. As visualized by a Venn diagram, five candidate genes were defined as hub genes (module genes) and DEGs in both CRC and IBD: These were the mutual genes that were upregulated in both the CRC and the IBD samples, each compared to the respective internal normal control tissue groups (Figure 5). Notably, despite the small number of genes, Reactome pathway analysis of these genes identified the antimicrobial peptide, IL-4, and IL-13 signaling, and metal sequestration by antimicrobial protein pathways as statistically significantly enriched (adjusted *p*-values < 0.05) **(** Supplementary Table S5 **)**. Notable, our identification of IL-4 and IL-13 signaling validated our previous identification of this pathway based on the most significant modules (Section 3.3 and Supplementary Table S4). Thus, our data further support the importance of IL-4 and IL-13 signaling in both CRC and IBD.

### 3.7 External Validation of Common Genes in CRC

**FIGURE 6:**
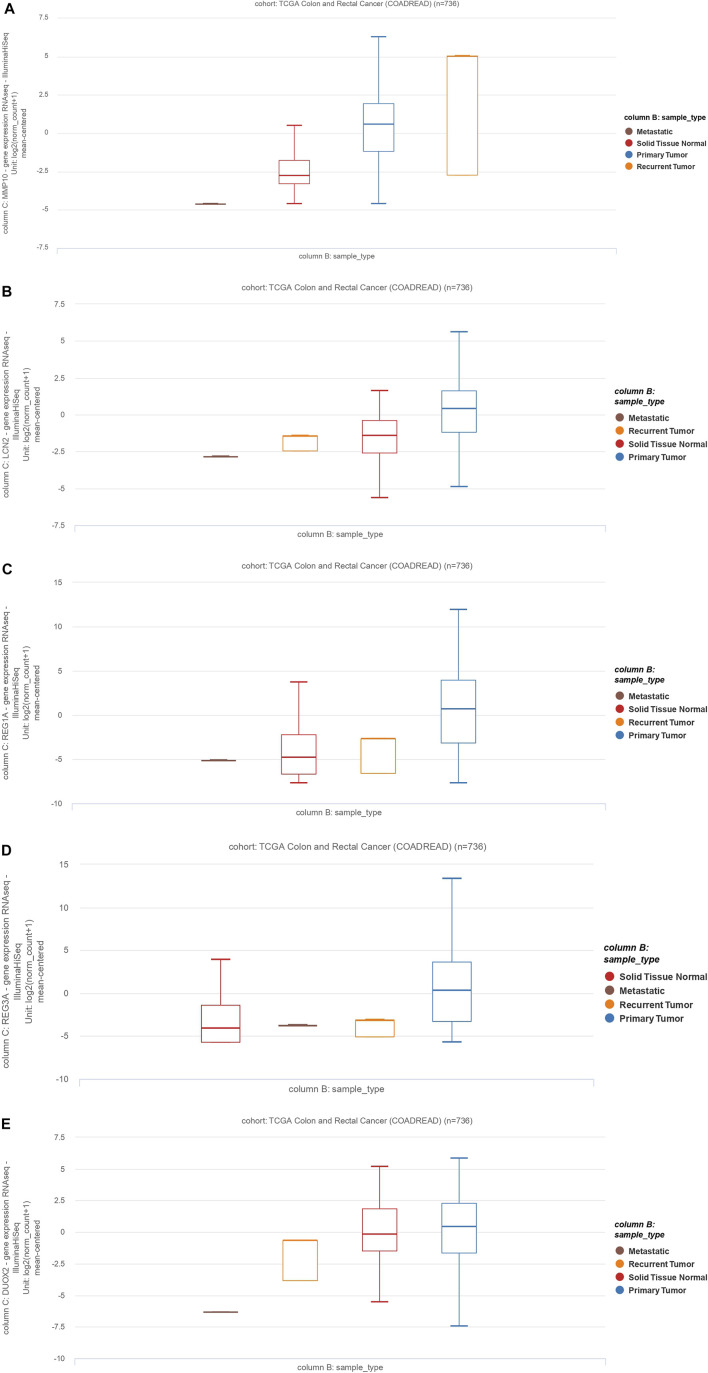
The *MMP10, LCN2, REG1A, REG3A,* and *DUOX2* expression profiles and clinicopathological data of colon adenocarcinoma cases and rectum adenocarcinoma (READ). Data are from The Cancer Genome Atlas (TCGA). **(A)**: *MMP10,* **(B)**: *LCN2,* **(C)**: *REG1A,* **(D)**: *REG3A,* **(E)**: *DUOX2*.

To further validate our *in-silico* study, we compared our results to TCGA Colon and Rectum adenocarcinoma data. We identified a total of five common genes (*MMP10, LCN2, REG1A, DUOX2,* and *REG3A*) whose expression was significantly upregulated. Here, we validate that these are significantly upregulated (*p* < 0.05) in various primary CRCs, recurrent CRCs, and metastatic CRCs compared to normal tissues (Figure 6). Thus, this validates our findings on an independent dataset from TCGA.

### 3.8 External Validation of Common Genes in IBD and its Clinical Impact

**FIGURE 7:**
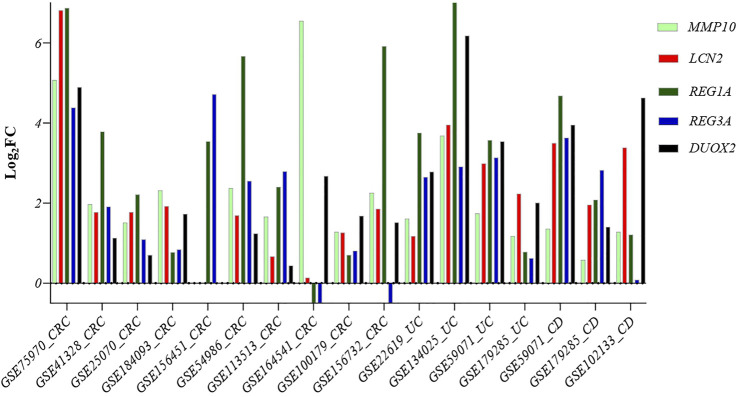
Comparison of the expression level of the five common genes in samples from different diseases: Crohn’s disease, ulcerative colitis, and CRC patients. Abbreviations: CRC: Colorectal cancer, UC: Ulcerative colitis, CD: Crohn’s disease.

To validate the role of our five identified genes signature in IBD and CRC, we also validated their gene expression levels in 15 independent datasets, including UC, CD, CRC, and corresponding controls (GSE75970, GSE41328, GSE25070, GSE184093, GSE156451, GSE54986, GSE113513, GSE164541, GSE100179, GSE156732, GSE22619, GSE134025, GSE59071, GSE179285, and GSE102133) (Supplementary Table S1 and Figure 7).

Our five identified genes signature was also significantly upregulated in these samples, suggesting a potential biomarker gene signature for identifying IBD patients at risk of developing CRC.

## 4 Discussion

Patients with inflammatory bowel disease (IBD), such as Crohn’s disease and ulcerative colitis, are at increased risk of developing colorectal cancer (CRC). Still, our knowledge about common and distinct pathways in these diseases remains elusive. Despite the reported correlation between IBD and CRC according to previous studies, some of the following research suggests otherwise (Söderlund et al., 2009; Jess et al., 2012b; Lutgens et al., 2013; Castaño-Milla et al., 2014). Despite disagreement regarding the specific incidence of CRC in patients with inflammatory bowel disease (IBD), the overall link between IBD and an elevated risk of developing CRC is well documented and widely accepted in clinical practice (Eaden et al., 2001; Jess et al., 2012a).

Therefore, we aimed to identify overlapping and divergent pathways in this study. We demonstrate that weighted gene co-expression network analysis (WGCNA) is suitable for this study. WGCNA is a systematic biological method used to characterize the patterns of gene connections between diverse datasets. This enables the identification of potential biomarker genes or therapeutic targets based on the interconnection of genes and the relationship between genes and clinicopathological features of patients (Liao et al., 2020).

In recent years, systems biology techniques, such as WGCNA, have been mainly used to analyze novel biomarkers in different diseases, such as adrenocortical cancer, breast cancer, and CRC (Keller et al., 2019; Shi et al., 2020). It has been reported that IBD is associated with an increased risk of increase risk of CRC development (Keller et al., 2019). However, work published to date has aimed to identify therapeutic targets for IBD or CRC individually, while shared and distinct genetic alterations between CRC and IBD–such as by using WGCNA–have not been a focus. Herein, we used WGCNA for the assessment of gene expression to describe hub modules and genes associated with the pathogenesis of IBD and CRC. In addition, we identified a signature of common significantly upregulated genes (*MMP10, LCN2, REG1A, DUOX2,* and *REG3A*), revealing the shared importance of IL-4 and IL-13 signaling pathway in both IBD and CRC.

Ulcerative colitis and Crohn’s disease are common forms of IBD. Ulcerative colitis is restricted to the mucosal surface of the colon, while Crohn’s disease may more broadly affect the gastrointestinal tract from the mouth to the anus with transmural inflammation (Kaistha and Levine, 2014). Our study has demonstrated that the most enriched pathways in IBD and CRC are receptor-ligand activity, system development, leukocyte migration, inflammatory response, and neutrophil chemotaxis. These pathways are primarily involved in the inflammation process, reinforcing that there is a correlation between IBD and CRC initiation.

Our work identifies five genes that are mutually overexpressed in IBD and CRC. As shown in Figure 5, *MMP10, LCN2, REG1A, REG3A,* and *DUOX2* are all upregulated genes in CRC and IBD patients compared to matched normal tissue samples. Notably, these five genes are from the shared modules and represent both hub genes and DEGs in CRC and IBD. As illustrated in Figure 6, the expression levels of the hub genes in each sample of selected datasets are significantly different between normal and patient samples, and this reveals the importance of IL-4 and IL-13 signaling in the pathogenesis of both IBD and CRC.

MMPs affect a wide range of immunological functions, including leukocyte infiltration and chemokine activity (Mcmahan et al., 2016). Many MMPs have been shown to be overexpressed in UC, and their increased expression levels have been associated with the exacerbated disease. MMP10 is produced predominantly by infiltrating myeloid cells in murine and human colitis. Bone marrow transplantation experiments showed that *MMP10* derived from bone marrow is involved in colitis severity (Koller et al., 2012). Furthermore, overexpression of *MMP10, MMP7,* and *MMP12* has been associated with the poor prognosis of CRC patients (Klupp et al., 2016). *MMP10* overexpression is also associated with the invasion of CRC cells (Klupp et al., 2016). Our results indicate that *MMP10* is differentially expressed in primary CRC, recurrent tumors, and metastatic tumors compared to normal tissue (Figure 6A).

Consistent with our work, as a member of the lipocalin superfamily, *LCN2* has been shown to play an essential role in the oncogenesis and progression of various tumors. Kim et al. demonstrated that *LCN2* is differentially expressed in CRC tissues compared to normal tissues (Kim et al., 2017). Similarly, Stallhofer et al. showed that *LCN2* expression is substantially increased in patients with active IBD compared to normal individuals (Stallhofer et al., 2015). Our bioinformatics results have shown that *LCN2* is differently expressed in primary CRC, recurrent tumors, and metastatic tumors compared to non-tumoral samples (Figure 6B).

*REG1A* is a type I subclass member of the Reg gene family. Astrosini et al. reported that *REG1A* is upregulated in early-stage CRC patients with poor clinical outcomes. Additionally, REG1A is a prognostic factor for CRC patients (Astrosini et al., 2008). Mao et al. demonstrated that *REG1A* was increased in the inflamed colorectal tissues of IBD-bearing patients. Additionally, abnormally expressed REG1A inhibited inflammatory responses, enhanced cell proliferation, and lowered epithelial apoptosis in intestinal epithelial cells (IEC). Mechanically, IL-6 and IL-22 induced a significant increase in *REG1A* transcription via activating the JAK/STAT3 signaling pathway. Additionally, the systematic introduction of *REG1A* lentivirus to mice significantly reduced DSS-induced inflammatory damage and preserved the integrity of the intestinal mucosal barrier. Collectively, their findings suggest that the new proliferative factor *REG1A*, which is regulated by IL-6/IL-22-JAK-STAT3 signaling, may represent an attractive therapeutic target for patients with inflammatory bowel disease (Mao et al., 2021).

Ying et al. have shown that increased expression of *REG3A* is associated with increased tumor size, poor tumor differentiation, low survival rate, and advanced tumor stage. Interestingly, *REG3A* knockdown substantially arrests tumor cells in the G1 phase of the cell cycle, inhibits cell proliferation, suppresses cell migration, and promotes apoptosis in CRC cells (Ye et al., 2016). Similarly, Du et al. have shown that single-chain variable fragment targeting REG3A (scFv-Reg3a) can inhibit cell proliferation and tumor migration in CRC. Finally, targeting REG3A enhances chemosensitivity to 5-fluorouracil in CRC (Du et al., 2020). In line with these observations, our results have shown that the expression of *REG1A* and *REG3A* is significantly increased in primary, recurrent, and metastatic CRC relative to non-tumor adjacent normal tissue (Figures 6C,D).

A fundamental gut-epithelial innate defense response is the release of hydrogen peroxide by dual NADPH oxidase (DUOX). *DUOX2* isoenzyme is the most induced gene in IBD, a disorder defined by an imbalanced gut microbiota-immune homeostasis (Grasberger et al., 2021). According to our analysis of datasets, we found out that this gene is also significantly upregulated in CRC and IBD tissues. In line with our results, Qi et al. have shown that expression of *DUOX2* is increased in CRC tissues.

Moreover, DUOX2 protein levels are substantially increased in stages II-IV CRC compared to stage I CRC tissues (Qi et al., 2016). Macfie et al. demonstrated that DUOX2 and DUOXA2 could form an enzymatic system to produce reactive oxygen species in active ulcerative colitis, and *DUOX2* is upregulated in active colitis (Macfie et al., 2014). Since dysregulated reactive oxygen species can damage the DNA, increased levels of reactive oxygen species have been associated with tumorigenicity (Derakhshani et al., 2021b).

Our pathway analysis using Reactome identified IL-4 and IL-13 signaling as significantly enriched pathways in IBD and CRC. The involvement of IL-4 has previously been reported in colitis and colorectal cancer (Jess et al., 2012a; Chung et al., 2019; Lin et al., 2019). IL-13 and IL-4 are structurally and functionally similar cytokines sharing common receptor subunits. They govern immunological responses and are also involved in developing a range of human neoplasms. Three distinct receptors have been discovered for IL-4; however, only IL-4 receptor type II (IL-4Rα/IL-13Rα1) is expressed in solid tumors. While IL-13 can also connect to three separate receptors, IL-13 receptor type I (IL-4Rα/IL-13Rα1/IL-13Rα2) and type II (IL-4Rα/IL-13Rα1) are expressed in solid tumors. After binding to its receptor, IL-4 and IL-13 can mediate tumor cell proliferation, survival, and metastasis in colorectal and gastric cancers (Song et al., 2021).

Exploiting systems biology to identify the common DEGs and pathways of CRC and IBD could provide the researchers with very important insights about the shared characteristics of these two nearly related diseases. This could lead to enhancing the prognosis of CRC in IBD patients and better treatment options.

## 5 Conclusion

Taken together, our results have shed new light on the molecular association between IBD and CRC. We identified a signature of five upregulated genes, *MMP10, LCN2, REG1A, REG3A*, and *DUOX2,* in both IBD and CRC. We also identified IL-4 and IL-13 signaling as the predominant shared pathway in IBD and CRC patients. These new insights demonstrated opportunities for the development of novel therapeutic strategies.

## Data Availability

The original contributions presented in the study are included in the article/Supplementary Material, further inquiries can be directed to the corresponding authors.

## Abbreviations

CRC, colorectal cancer; IBD, inflammatory bowel disease; WGCNA, weighted network correlation analysis; KEGG, kyoto encyclopedia of genes and genomes; DEGs, differentially expressed genes; MM, module membership; GS, gene significance; GEO, gene expression omnibus; GO, gene ontology; ME, module eigengene; scFv-Reg3a, single-chain variable fragment targeting REG3A; TOM, topological overlap matrix; dissTOM, dissimilarity TOM.
