# Supplementary material for: Identification of Common and Distinct Pathways in Inflammatory Bowel Disease and Colorectal Cancer: A Hypothesis Based on Weighted Gene Co-Expression Network Analysis
Source: Front Genet. 2022 Mar 31;13:848646. doi: 10.3389/fgene.2022.848646 (PMC9008839; doi:10.3389/fgene.2022.848646)
Supplement: Supplementary file 1 [file DataSheet1.docx]

**Supplementary Tables:**

**Table S1.** Characteristics of the specimens in each dataset (<https://www.ncbi.nlm.nih.gov/geo/> ).

| Number | GEO accession | Experiment type | Tissue | Disease | Samples | Platforms | Country |
| --- | --- | --- | --- | --- | --- | --- | --- |
| 1 | GSE110224 | Expression profiling by array | Colorectal Tumor and normal adjacent | CRC | 34 | GPL570 | Greece |
| 2 | GSE4183 | Expression profiling by array | Colonic biopsies of patients with CRC, IBD, and healthy normal controls | CRC and IBD | 53 | GPL570 | Hungary |
| 3 | GSE75970 | Expression profiling by array | Colorectal Tumor and paraneoplastic tissue | CRC | 8 | GPL14550 | China |
| 4 | GSE41328 | Expression profiling by array | Colon adenocarcinoma and matched normal colon tissue | CRC | 20 | GPL570 | USA |
| 5 | GSE25070 | Expression profiling by array | Colorectal tumors and matched histologically normal adjacent | CRC | 52 | GPL6883 | USA |
| 6 | GSE184093 | Expression profiling by array | Colorectal cancer and histologically normal tissue adjacent to the tumor | CRC | 18 | GPL20115 | China |
| 7 | GSE156451 | Expression profiling by high throughput sequencing | Tumor and native tissue from CRC patients | CRC | 144 | GPL24676 | China |
| 8 | GSE54986 | Expression profiling by array | Cancerous and paracancerous tissues | CRC | 12 | GPL10558 | China |
| 9 | GSE113513 | Expression profiling by array | Colorectal carcinoma tissue and non-cancerous surrounding tissue | CRC | 28 | GPL15207 | China |
| 10 | GSE164541 | Expression profiling by high throughput sequencing | Primary CRC, adenoma, and adjacent normal tissue | CRC | 15 | GPL16791 | China |
| 11 | GSE100179 | Expression profiling by array | Colorectal adenoma, colorectal cancer, and healthy colonic tissues | CRC | 60 | GPL17586 | Hungary |
| 12 | GSE156732 | Expression profiling by array | colorectal cancer and adjacent normal tissues | CRC | 12 | GPL26963 | China |
| 13 | GSE22619 | Expression profiling by array | Ulcerative colitis and primary mucosal tissue (healthy) | Ulcerative colitis | 20 | GPL570 | Germany |
| 14 | GSE134025 | Expression profiling by array | Ulcerative colitis and healthy control | Ulcerative colitis | 6 | GPL20115 | China |
| 15 | GSE59071 | Expression profiling by array | Ulcerative colitis, Crohn's disease patients, healthy controls | Ulcerative colitis and Crohn's disease | 116 | GPL6244 | Belgium |
| 16 | GSE179285 | Expression profiling by array | Crohn's disease, ulcerative colitis, and normal healthy controls | Ulcerative colitis and Crohn's disease | 254 | GPL6480 | USA |
| 17 | GSE102133 | Expression profiling by array | Ileal mucosa from Crohn's disease patients, healthy controls | Crohn's disease | 77 | GPL6244 | Belgium |

**Table S2:** Module color characteristics of the CRC dataset (GSE110224). The co-expression modules were identified by WGCNA. The Grey module, including 258 genes, failed to classify as a distinct co-expression module. Hence, this module was eliminated from further analysis. P-values less than 0.05 are considered statistically significant.

| Module color | Cor | P-value | Gene |
| --- | --- | --- | --- |
| Blue | -0.94 | 4.00E-11 | 447 |
| Turquoise | 0.94 | 2.00E-10 | 1112 |
| Tan | 0.88 | 7.00E-08 | 126 |
| Royal blue | -0.86 | 4.00E-07 | 80 |
| Brown | -0.81 | 4.00E-06 | 359 |
| Light yellow | 0.77 | 3.00E-05 | 89 |
| Pink | 0.73 | 1.00E-04 | 163 |
| Salmon | -0.72 | 1.00E-04 | 119 |
| Yellow | 0.73 | 1.00E-04 | 333 |
| Red | 0.72 | 2.00E-04 | 187 |
| Purple | -0.7 | 3.00E-04 | 138 |
| Green | -0.66 | 7.00E-04 | 294 |
| Green yellow | 0.55 | 0.008 | 128 |
| Magenta | 0.55 | 0.008 | 163 |
| Orange | -0.54 | 0.01 | 41 |
| Light green | 0.44 | 0.04 | 95 |
| Midnight blue | -0.42 | 0.05 | 110 |
| Light cyan | -0.41 | 0.06 | 108 |
| Dark red | -0.4 | 0.07 | 77 |
| Dark grey | -0.3 | 0.2 | 51 |
| Grey60 | -0.31 | 0.2 | 96 |
| Cyan | -0.25 | 0.3 | 117 |
| Dark turquoise | -0.18 | 0.4 | 61 |
| Black | -0.055 | 0.8 | 172 |
| Grey | 0.053 | 0.8 | 258 |
| Dark green | 0.036 | 0.9 | 76 |

**Table S3:** Module color characteristics of the IBD dataset (GSE4183). The co-expression modules were identified by WGCNA. The Grey module, including 867 genes, failed to classify as a distinct co-expression module. Thus, this module was eliminated from further analysis. P-values less than 0.05 are considered statistically significant.

| Module color | Cor | P-value | Gene |
| --- | --- | --- | --- |
| Turquoise | 0.91 | 4.00E-08 | 1463 |
| Blue | 0.81 | 2.00E-05 | 1259 |
| Brown | -0.66 | 0.002 | 539 |
| Green | 0.34 | 0.1 | 298 |
| Yellow | 0.38 | 0.1 | 429 |
| Red | -0.0023 | 1 | 145 |
| Grey | -0.075 | 0.8 | 867 |

| **Table S4. Identification of 48 hub genes shared between CRC and IBD** (blue and turquoise modules from each dataset) | | |
| --- | --- | --- |
| **Gene Name** | **NCBI Entrez Gene ID** | **Description** |
| AADACL2 | 344752 | arylacetamide deacetylase like 2(AADACL2) |
| ANXA9 | 8416 | annexin A9(ANXA9) |
| ASGR1 | 432 | Asialoglycoprotein receptor 1(ASGR1) |
| BFSP1 | 631 | beaded filament structural protein 1(BFSP1) |
| C16ORF89 | 146556 | chromosome 16 open reading frame 89(C16orf89) |
| C1QTNF2 | 114898 | C1q and tumor necrosis factor related protein 2(C1QTNF2) |
| C2ORF40 (ECRG4) | 84417 | chromosome 2 open reading frame 40(C2orf40) |
| C7 | 730 | complement C7(C7) |
| CCL20 | 6364 | C-C motif chemokine ligand 20(CCL20) |
| CDA | 978 | cytidine deaminase (CDA) |
| CLDN1 | 9076 | claudin 1(CLDN1) |
| CLSTN2 | 64084 | Calsyntenin 2(CLSTN2) |
| DUOX2 | 50506 | dual oxidase 2(DUOX2) |
| DUOXA2 | 405753 | dual oxidase maturation factor 2(DUOXA2) |
| ERVMER34-1 | 100288413 | endogenous retrovirus group MER34 member 1(ERVMER34-1) |
| FAM19A2 (TAFA2) | 338811 | family with sequence similarity 19 member A2, C-C motif chemokine like (FAM19A2) |
| FAM3B | 54097 | family with sequence similarity 3 member B(FAM3B) |
| FCN3 | 8547 | ficolin 3(FCN3) |
| FEV | 54738 | FEV, ETS transcription factor (FEV) |
| GRAMD2 (GRAMD2A) | 196996 | GRAM domain containing 2(GRAMD2) |
| GREM2 | 64388 | gremlin 2, DAN family BMP antagonist (GREM2) |
| HYAL3 | 8372 | Hyaluronoglucosaminidase 3(HYAL3) |
| IL13RA2 | 3598 | interleukin 13 receptor subunit alpha 2(IL13RA2) |
| IL17A | 3605 | interleukin 17A(IL17A) |
| KLHDC1 | 122773 | Kelch domain containing 1(KLHDC1) |
| LCN2 | 3934 | lipocalin 2(LCN2) |
| LGR5 | 8549 | leucine rich repeat containing G protein-coupled receptor 5(LGR5) |
| LINC00920 | 100505865 | long intergenic non-protein coding RNA 920(LINC00920) |
| LOC101927746 (EMSLR) | 101927746 | uncharacterized LOC101927746(LOC101927746) |
| LOC340090 | 340090 | uncharacterized LOC340090(LOC340090) |
| LRRC20 | 55222 | leucine rich repeat containing 20(LRRC20) |
| MFAP4 | 4239 | microfibrillar associated protein 4(MFAP4) |
| MMP10 | 4319 | matrix metallopeptidase 10(MMP10) |
| MMP9 | 4318 | matrix metallopeptidase 9(MMP9) |
| NOS3 | 4846 | nitric oxide synthase 3(NOS3) |
| NUP62CL | 54830 | nucleoporin 62 C-terminal like (NUP62CL) |
| PDZK1IP1 | 10158 | PDZK1 interacting protein 1(PDZK1IP1) |
| PF4 | 5196 | platelet factor 4(PF4) |
| PRPH | 5630 | Peripherin (PRPH) |
| RDH5 | 5959 | retinol dehydrogenase 5(RDH5) |
| REG1A | 5967 | regenerating family member 1 alpha (REG1A) |
| REG3A | 5068 | regenerating family member 3 alpha (REG3A) |
| ROBO3 | 64221 | roundabout guidance receptor 3(ROBO3) |
| SALL4 | 57167 | spalt like transcription factor 4(SALL4) |
| SERPINF2 | 5345 | serpin family F member 2(SERPINF2) |
| SLC6A20 | 54716 | solute carrier family 6 member 20(SLC6A20) |
| SPNS3 | 201305 | sphingolipid transporter 3 (putative)(SPNS3) |
| TCAM1P | 146771 | testicular cell adhesion molecule 1, pseudogene (TCAM1P) |

| **Table S5. Enriched pathways identified by Reactome (based on 48 hub genes)** | | | | | | | | | |
| --- | --- | --- | --- | --- | --- | --- | --- | --- | --- |
|  | **Entities** |  |  |  |  |  | **Reactions** | | |
| **Pathway name** | **found** | **Total** | **ratio** | **pValue** | **FDR** | **FDR summary** | **found** | **total** | **ratio** |
| Interleukin-4 and Interleukin-13 signaling | 8 | 211 | 0.015 | 2.25E-06 | 0.0003 | *** | 9 | 47 | 0.003 |
| Signaling by Interleukins | 10 | 643 | 0.044 | 2.45E-04 | 0.0162 | * | 11 | 493 | 0.036 |
| Common Pathway of Fibrin Clot Formation | 2 | 25 | 0.002 | 4.74E-03 | 0.2040 | n.s. | 1 | 29 | 0.002 |
| Activation of Matrix Metalloproteinases | 2 | 35 | 0.002 | 9.05E-03 | 0.2040 | n.s. | 6 | 27 | 0.002 |
| Cytokine Signaling in Immune system | 10 | 1,092 | 0.075 | 1.19E-02 | 0.2040 | n.s. | 11 | 708 | 0.052 |
| Variant SLC6A20 contributes towards hyperglycinuria (HG) and iminoglycinuria (IG) | 1 | 3 | 0 | 1.21E-02 | 0.2040 | n.s. | 1 | 1 | 0 |
| Formation of Fibrin Clot (Clotting Cascade) | 2 | 43 | 0.003 | 1.34E-02 | 0.2040 | n.s. | 1 | 61 | 0.005 |
| Transcriptional regulation of pluripotent stem cells | 2 | 45 | 0.003 | 1.46E-02 | 0.2040 | n.s. | 6 | 35 | 0.003 |
| Chemokine receptors bind chemokines | 2 | 57 | 0.004 | 2.27E-02 | 0.2480 | n.s. | 2 | 19 | 0.001 |
| NOSIP mediated eNOS trafficking | 1 | 7 | 0 | 2.80E-02 | 0.2480 | n.s. | 2 | 2 | 0 |
| Terminal pathway of complement | 1 | 8 | 0.001 | 3.19E-02 | 0.2480 | n.s. | 4 | 5 | 0 |
| Collagen degradation | 2 | 69 | 0.005 | 3.22E-02 | 0.2480 | n.s. | 10 | 34 | 0.003 |
| NOSTRIN mediated eNOS trafficking | 1 | 10 | 0.001 | 3.98E-02 | 0.2480 | n.s. | 4 | 4 | 0 |
| RUNX1 regulates genes involved in megakaryocyte differentiation and platelet function | 2 | 78 | 0.005 | 4.02E-02 | 0.2480 | n.s. | 2 | 33 | 0.002 |
| ROBO receptors bind AKAP5 | 1 | 11 | 0.001 | 4.37E-02 | 0.2480 | n.s. | 3 | 7 | 0.001 |
| Regulation of commissural axon pathfinding by SLIT and ROBO | 1 | 12 | 0.001 | 4.75E-02 | 0.2480 | n.s. | 4 | 5 | 0 |
| Interaction With Cumulus Cells and The Zona Pellucida | 1 | 12 | 0.001 | 4.75E-02 | 0.2480 | n.s. | 1 | 2 | 0 |
| Ficolins bind to repetitive carbohydrate structures on the target cell surface | 1 | 12 | 0.001 | 4.75E-02 | 0.2480 | n.s. | 1 | 3 | 0 |
| Interleukin-10 signaling | 2 | 86 | 0.006 | 4.79E-02 | 0.2480 | n.s. | 1 | 15 | 0.001 |

| **Table S6. Enriched pathways identified by Reactome (based on 4 enriched genes).** | | | | | | | | | |
| --- | --- | --- | --- | --- | --- | --- | --- | --- | --- |
|  | **Entities** |  |  |  |  |  | **Reactions** | | |
| **Pathway name** | **found** | **Total** | **ratio** | **pValue** | **FDR** | **FDR summary** | **found** | **total** | **ratio** |
| Antimicrobial peptides | 2 | 123 | 0.008 | 1.46E-03 | 0.0307 | * | 5 | 58 | 0.004 |
| Interleukin-4 and Interleukin-13 signaling | 2 | 211 | 0.015 | 4.21E-03 | 0.0421 | * | 1 | 47 | 0.003 |
| Metal sequestration by antimicrobial proteins | 1 | 13 | 0.001 | 6.24E-03 | 0.0437 | * | 1 | 5 | 0 |
| Thyroxine biosynthesis | 1 | 27 | 0.002 | 1.29E-02 | 0.0646 | n.s. | 1 | 11 | 0.001 |
| Activation of Matrix Metalloproteinases | 1 | 35 | 0.002 | 1.67E-02 | 0.0669 | n.s. | 4 | 27 | 0.002 |
| Metabolism of amine-derived hormones | 1 | 56 | 0.004 | 2.66E-02 | 0.0691 | n.s. | 1 | 21 | 0.002 |
| Collagen degradation | 1 | 69 | 0.005 | 3.27E-02 | 0.0691 | n.s. | 3 | 34 | 0.003 |
| Signaling by Interleukins | 2 | 643 | 0.044 | 3.54E-02 | 0.0691 | n.s. | 1 | 493 | 0.036 |
| Iron uptake and transport | 1 | 83 | 0.006 | 3.93E-02 | 0.0691 | n.s. | 3 | 34 | 0.003 |
| MAPK6/MAPK4 signaling | 1 | 106 | 0.007 | 4.99E-02 | 0.0691 | n.s. | 1 | 40 | 0.003 |
| Degradation of the extracellular matrix | 1 | 148 | 0.01 | 6.91E-02 | 0.0691 | n.s. | 11 | 105 | 0.008 |
| Cytokine Signaling in Immune system | 2 | 1,092 | 0.075 | 9.19E-02 | 0.0919 | n.s. | 1 | 708 | 0.052 |
| Immune System | 3 | 2,681 | 0.184 | 1.22E-01 | 0.1220 | n.s. | 7 | 1,623 | 0.12 |
| Innate Immune System | 2 | 1,334 | 0.092 | 1.30E-01 | 0.1300 | n.s. | 6 | 710 | 0.053 |
| Extracellular matrix organization | 1 | 329 | 0.023 | 1.48E-01 | 0.1480 | n.s. | 11 | 319 | 0.024 |
| MAPK family signaling cascades | 1 | 380 | 0.026 | 1.69E-01 | 0.1690 | n.s. | 1 | 122 | 0.009 |
| Neutrophil degranulation | 1 | 480 | 0.033 | 2.09E-01 | 0.2090 | n.s. | 1 | 10 | 0.001 |
| Metabolism of amino acids and derivatives | 1 | 661 | 0.045 | 2.78E-01 | 0.2780 | n.s. | 1 | 285 | 0.021 |
| Transport of small molecules | 1 | 958 | 0.066 | 3.79E-01 | 0.3790 | n.s. | 3 | 442 | 0.033 |
| Signal Transduction | 1 | 2,993 | 0.206 | 8.01E-01 | 0.8010 | n.s. | 1 | 2,445 | 0.181 |

n.s: not significant. FDR: false discovery rate

**Supplementary figures:**

**Figure S1:**


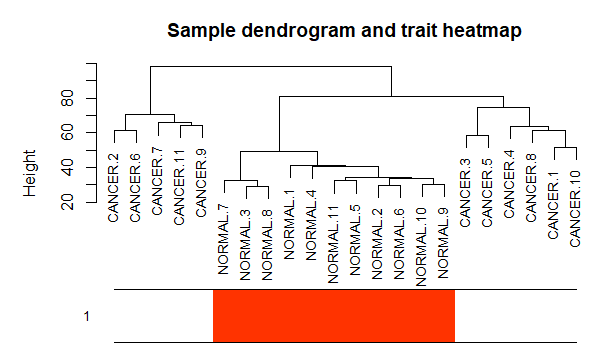

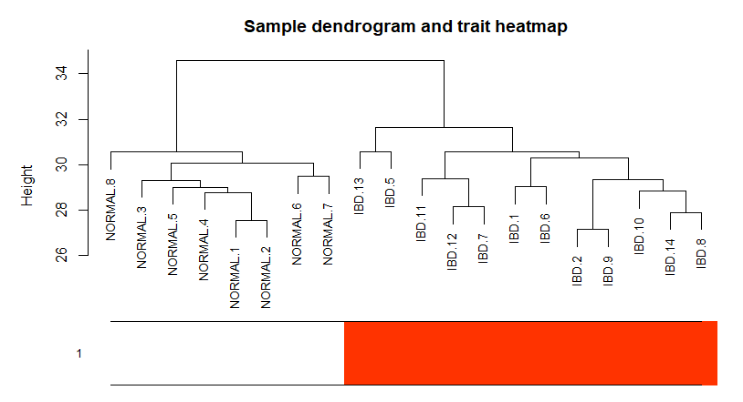


**GSE110224**

**GSE4183**

**Figure S1:** Sample cluster analysis based on mRNA data (GSE110224 (CRC dataset) and GSE4183 (IBD dataset)). The sample dendrogram visualizes how the samples cluster and identifies any obvious outliers. Inflammatory bowel disease (IBD).

**Figure S2:**


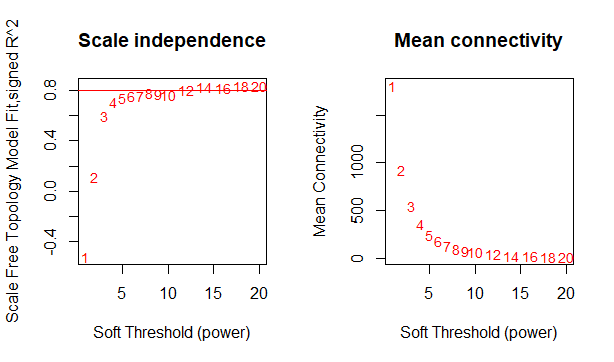


**GSE110224**


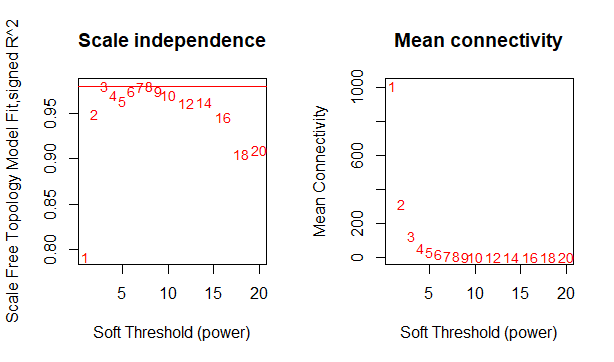


**GSE4183**

**Figure S2:** Determination of the soft threshold using the WGCNA algorithm. The approximate scale-free fit index can be attained at the soft-thresholding powers of 12 and 7 (GSE110224 and GSE4183, respectively).

**Figure S3:**

**GSE110224**

**GSE4183**


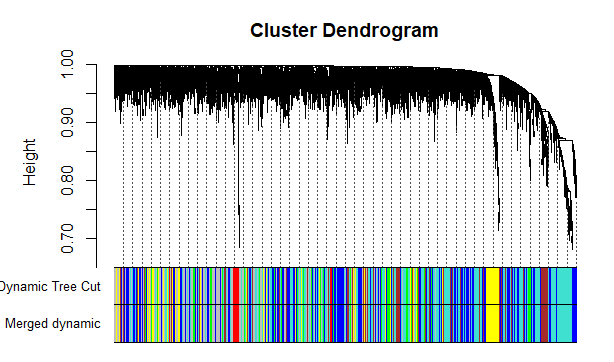

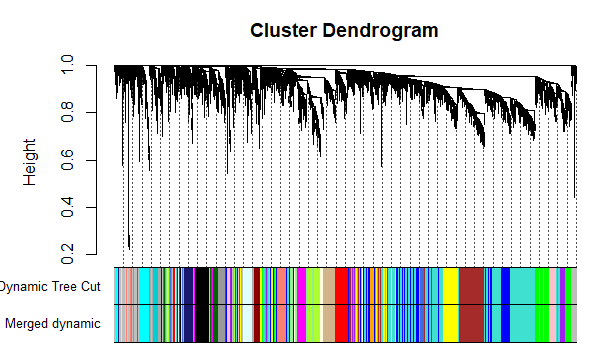


**Figure S3:** Cluster dendrogram and module assignment from WGCNA for CRC (GSE110224, left) and IBD (GSE4183, right). The branches correspond to highly interconnected groups of genes. Colors in the horizontal bar represent the modules.

**Figure S4:**


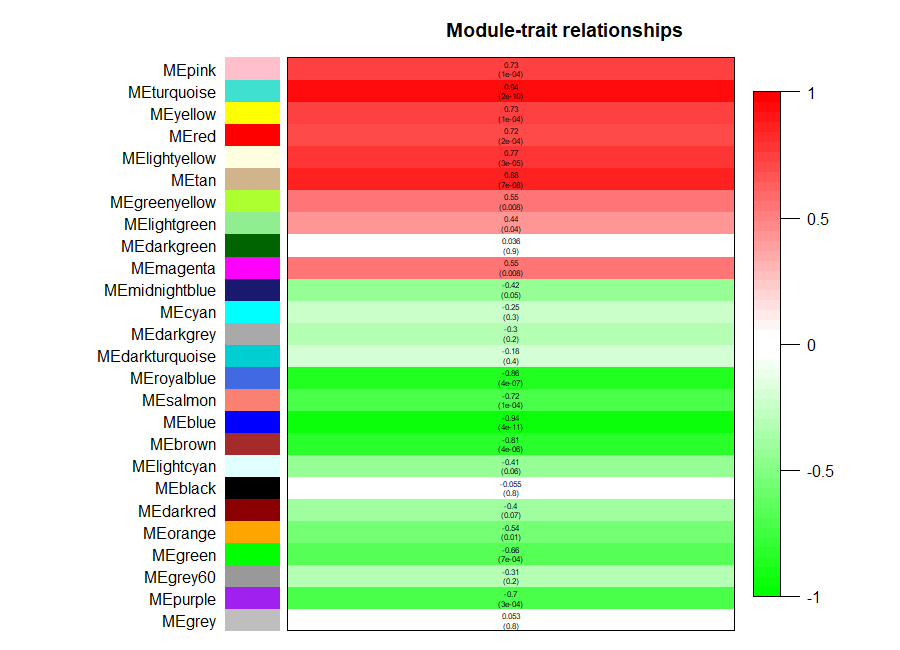

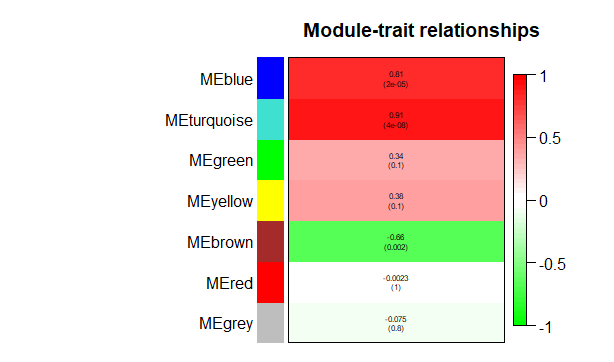


**GSE110224**

**GSE4183**

**Figure S4:** Module-trait relationship and enrichment of interest module. Individual rows correspond to module eigengenes, and columns match disease status. Numbers indicate the corresponding correlation and p-value in each cell. The more positively correlated the module and the disease (red), the more negatively correlated (green).

**Figure S5:**


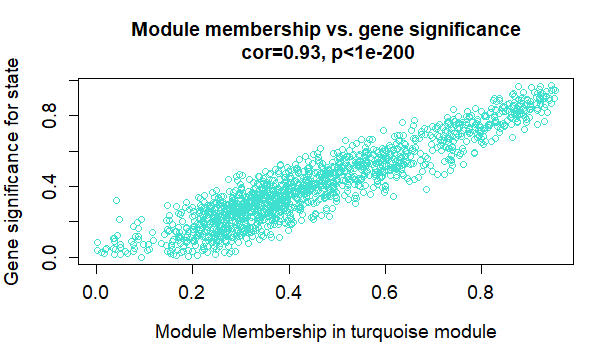

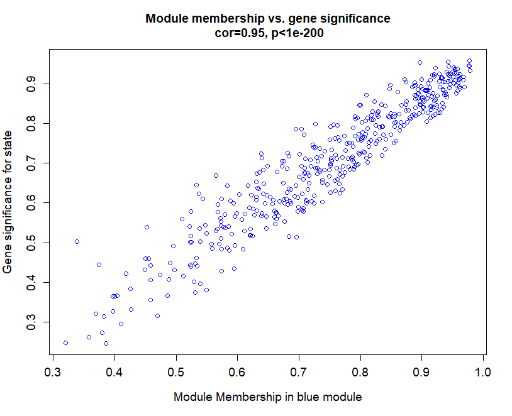


**GSE110224**

**GSE4183**

**Figure S5:** Module features of gene significance (GS) and module membership (MM). Modules significantly correlated with H status (control vs. patient). Each point represents an individual gene within each module, which are plotted by GS on the y-axis and MM on the x-axis.
